# BET Bromodomain Inhibitor iBET151 Impedes Human ILC2 Activation and Prevents Experimental Allergic Lung Inflammation

**DOI:** 10.3389/fimmu.2019.00678

**Published:** 2019-04-09

**Authors:** Bernhard Kerscher, Jillian L. Barlow, Batika M. Rana, Helen E. Jolin, Mayuri Gogoi, Michelle A. Bartholomew, Deepali Jhamb, Ashutosh Pandey, David F. Tough, Antoon J. M. van Oosterhout, Andrew N. J. McKenzie

**Affiliations:** ^1^Medical Research Council, Laboratory of Molecular Biology, Cambridge, United Kingdom; ^2^Allergic Inflammation DPU, Respiratory Therapy Area, GlaxoSmithKline, Medicines Research Centre, Stevenage, United Kingdom; ^3^Computational Biology, GSK R&D, Collegeville, PA, United States; ^4^Epigenetics DPU, Immunoinflammation Therapy Area Unit, Glaxo Smith Kline, Medicines Research Centre, Stevenage, United Kingdom

**Keywords:** ILC2, Th2, asthma, allergy, bromodomain, extra-terminal motif protein

## Abstract

Group 2 innate lymphoid cells (ILC2) increase in frequency in eczema and allergic asthma patients, and thus represent a new therapeutic target cell for type-2 immune-mediated disease. The bromodomain and extra-terminal (BET) protein family of epigenetic regulators are known to support the expression of cell cycle and pro-inflammatory genes during type-1 inflammation, but have not been evaluated in type-2 immune responses. We isolated human ILC2 and examined the capacity of the BET protein inhibitor, iBET151, to modulate human ILC2 activation following IL-33 stimulation. iBET151 profoundly blocked expression of genes critical for type-2 immunity, including type-2 cytokines, cell surface receptors and transcriptional regulators of ILC2 differentiation and activation. Furthermore, *in vivo* administration of iBET151 during experimental mouse models of allergic lung inflammation potently inhibited lung inflammation and airways resistance in response to cytokine or allergen exposure. Thus, iBET151 effectively prevents human ILC2 activation and dampens type-2 immune responses.

## Introduction

Innate lymphoid cells (ILCs) have emerged as strategic players in inflammation and immunity, and represent new therapeutic targets in disease (1, 2). ILCs have been divided into five subsets based on their development and function: NK cells, ILC1s, ILC2s, ILC3s, and LTi cells (3). ILC2s were first described as playing important roles in the initiation of inflammation at mucosal barrier surfaces in response to infection or tissue damage (1, 4). However, it is now apparent that ILC2s can play more complex roles throughout the duration of immune responses, participating in the transition from innate to adaptive immunity and contributing to chronic inflammation. The proximity of ILC2s to epithelial surfaces and their constitutive strategic positioning in other tissues throughout the body ensures that, in spite of their rarity, ILC2s are able to regulate immune homeostasis and respond rapidly to damage or infection.

ILC2s are key drivers of allergic disease and upon activation express very high levels of IL-5, IL-6, and IL-13, but also secrete many other cytokines, including IL-4, GM-CSF, IL-9, and IL-10. These type-2 cytokine-expressing ILCs express receptors for epithelium-derived cytokines such as IL-25, IL-33, and TSLP required for their activation, but also depend on signals from common gamma chain cytokines including IL-2 or IL-7. It is the combination of these factors and signals transmitted by cell surface receptors such as ICOS, PD-1, ICAM-1, MHCII, OX40L that provide micro-environmental cues that lead to subtly different ILC2 subtypes in different tissues during homeostasis and in response to the variety of inflammatory stimuli encountered (5–9). Interestingly, genome-wide association studies of asthma have identified RORα (a critical transcription factor for ILC2 development), as well as TSLP, IL-33 and the IL33-receptor (IL1RL1, ST2) as asthma susceptibility genes, supporting the importance of this pathway for asthma.

In mouse models, lung-resident ILC2s contributed to airway hyperreactivity, induced by viral or allergen challenge (10). Human ILC2s were reported in lung parenchyma and bronchoalveolar lavage (BAL) fluid, and defined by Spits and colleagues as lineage negative CD45^hi^CD127^+^CD117^+^CRTH2^+^CD161^+^ cells that were also present in peripheral blood, fetal gut and the inflamed nasal polyps of patients with rhino-sinusitis (11). These cells responded to epithelium-derived cytokines by producing large amounts of IL-5 and IL-13. ILC2s have been shown to increase in frequency in allergic asthma patients as compared to healthy controls (12–16), and it has been reported that exposure of ILC2s to TSLP leads to their becoming steroid resistant (14). ILC2s were also found to be resident in human skin, enriched in atopic dermatitis (eczema) lesions (17), and responsive to IL-33, IL-25, TSLP, and also to the CRTH2 ligand PGD_2_ (18). More recently it has been reported that ILC2 also retain a degree of plasticity and can produce IFNγ or IL-17A dependent on the local cytokine environment in inflamed tissues (19, 20).

Dysregulation of ILC2 function contributes to chronic pathologies such as allergy, autoimmunity and inflammation (10). Consequently, ILC2s represent a new opportunity for therapeutic intervention in diseases including allergy and asthma. To realize this potential a better understanding of mouse and human ILC populations and biology, and their tractability to prospective therapies, is required.

BET domain-containing protein family (Brd2, Brd3, Brd4, and Brdt) are epigenetic ‘reader’ proteins that play important roles in controlling expression of cell cycle and pro-inflammatory genes (21). BET proteins bind to acetylated lysine molecules via N-terminal bromodomains, with each family member possessing two of these domains. Cell signaling-induced acetylation of histone tails and transcription factors leads to BET protein localization at gene enhancers and promoters, where they act to regulate gene expression. BET-bromodomain inhibitors (e.g., iBET151) that block the acetyl-lysine binding pocket of the BET proteins have been demonstrated to be beneficial in a number of type-1 inflammatory disease models (22–24), but little is known about their ability to modulate allergic disease.

We examined the capacity of iBET151 to block the activation of human ILC2s following IL-33 stimulation. iBET151 profoundly blocked expression of a suite of genes that are critical for type-2 immunity, including type-2 cytokines, cell surface receptors, (including ICOS, CD40 ligand (CD40L/CD154), ICAM1, and chemokine receptors), and transcriptional regulators of ILC2 differentiation and activation. Furthermore, *in vivo* administration of iBET151 potently inhibited lung inflammation induced by cytokine or allergen exposure. Thus, iBET151 can block human ILC2 activation and dampen type-2 responses in experimental mouse models of allergic lung inflammation.

## Materials and Methods

### Mice

Wildtype C57BL/6 mice and *Il33*-citrine (*Il33*^*cit*/+^) reporter mice (25) were bred inhouse. All mice were maintained in the Medical Research Council (MRC) ARES animal facility under specific pathogen-free conditions.

### iBET151

Lyophilized iBET151 (GSK1210151A) was dissolved in DMSO to generate a 150 mM stock solution and stored at−20°C. For *in vivo* experiments, iBET stock solution was diluted in PBS (pH ~5.5) containing 10% kleptose.

### Isolation of Human ILC

Lymphocytes were enriched from 150 ml human peripheral blood using lymphoprep (Axis-Shield) according to the manufacturer's instructions. The collected PBMC fraction was washed with PBS/2% FCS followed by red blood cell lysis with ACK solution for 5 min at room temperature. To pre-enrich ILCs, cells were stained with biotinylated anti-CD3, CD14 and B220 in the presence of TruStain FcX (anti-CD16/CD32) followed by depletion with M280 streptavidin beads (Invitrogen). Subsequently, the enriched fraction was stained with CD45-AF488, CD127-PE-Cy7, CD117-PE, CRTH2-BV421, a lineage cocktail (APC-streptavidin, CD8a, CD11b, CD11c, FcεRIα, CD123, CD20, CD56, CD71) and Draq7-viability dye, followed by sorting of ILCs as indicated in Supplementary Figure S1 on an Influx FACS. Sorted ILC2s were cultured in RPMI (GIBCO) supplemented with 10% FCS, 100 μM 2-mercaptoethanol, 100 μg/ml penicillin, 100 IU/ml streptomycin, 20 mM HEPES and 10 ng/ml recombinant human IL-2, IL-7 and IL-33 (as indicated).

### *Ex vivo* Stimulated Human ILC2s

ILC2s designated as Draq7^−^(live cells)CD45^+^Lin^−^(streptavidin-APC, CD8a, CD11b, CD11c, FcεRIα, CD123, CD20, CD56, CD71)CD127^+^CRTH2^+^ cells, were purified by flow cytometry from the peripheral blood of 3 individual donors and cultured separately for 24 h with IL-2, IL-7, and with or without IL-33 (all at 10 ng/ml). 250 nM iBET151 was added to some wells as indicated. Cells were pelleted and RNA prepared for RNAseq analysis.

### *In vitro* Cultured Cells

Draq7^−^(live cells)CD45^+^Lin^−^(streptavidin-APC, CD8a, CD11b, CD11c, FcεRIα, CD123, CD20, CD56, CD71)CD127^+^CRTH2^+^ ILC2s were isolated from the peripheral blood of 3 individual donors and cultured separately in cycling cultures. Purified human ILC2s were grown in media containing IL-2 and IL-7 (both at 10 ng/ml) with IL-33 (10 ng/ml) added to the cultures every alternate week. ILC2s were then rested for 3 days in IL-2 and IL-7-containing media (no IL-33) before being divided and added to the following culture conditions: IL-2/IL-7, +/−IL-33 (all at 10 ng/ml), +/− 125 nM iBET151 for 24 h. Each sample group only includes genes with an expression of >5RPKM.

Mouse ILC2s were purified from IL-33-treated lungs (see methods for IL-33-induced type 2 lung inflammation) as LiveCD45^+^Lineage^−^IL-7Rα^+^ICOS^+^ and cultured for 24 hrs with IL-7 (10 ng/ml) and with or without IL-33 (10 ng/ml). Mouse alveolar macrophages were purified from naïve lungs as LiveCD45^+^F4/80^+^ and cultured for 24 hrs with LPS (100 ng/ml). iBET151 was added to wells at 250, 125, or 62.5 nM. Golgi stop was added to the culture for the last 4 hrs and cells were then subjected to flow cytometric analysis.

### Flow Cytometric Analyses

Bronchoalveolar lavage (BAL) was collected by flushing the lungs with 1 ml PBS, retrieved volumes recorded, cells pelleted, and supernatants frozen at −20°C. Red blood cell lysis was performed on the pellet in ACK solution followed by staining for flow cytometry. Lungs were mechanically dissociated in RPMI-1640 (10 mM HEPES), and digested with collagenase I (500 U/ml, Life Technologies) and DNase I (0.2 mg/ml, Roche) at 37°C whilst shaking. Digested tissues were passed through a 70 μm cell strainer, connective tissue removed by centrifugation on a 25% Percoll gradient and red blood cells lysed in ACK solution for 5 min. Single cell suspensions were stained, washed and analyzed in PBS containing 2% FCS and 2 mM EDTA.

Cell suspensions were incubated with anti-mouse CD16/32 (BioXCell) to block Fc receptors and stained as indicated. Antibodies were purchased from eBioscience, Biolegend and BD and included: B220, CD3, CD4, CD5, CD8, CD11b, CD11c, CD19, CD24, CD45, CD127, Epcam, FcεR1α, GATA3, Gr1, ICOS, IL-13, IL-5, Ly6G, MHCII, NK1.1, SiglecF, and ST2. The lineage cocktail contained the antibodies CD3, CD5, CD11b, CD11c, CD19, F4/80, FcεR1α, Gr1, NK1.1, TCRβ, and Ter119, if not included separately in the panel. Live cells were identified using eFluor viability dyes (eBioscience) and cell numbers assessed with Precision Count Beads (Biolegend).

Cells were acquired on a BD Fortessa flow cytometry instrument and data analyzed using FlowJo (version 9.9). Myeloid cells were defined as eosinophils (SiglecF^+^, CD11c^−^), neutrophils (CD11b^+^, Ly6G^+^), alveolar macrophages (CD11c^+^, SiglecF^+^), interstitial macrophages (CD11b^+^, F4/80^+^), and DC (CD11c^+^, MHCII^+^). Lymphoid cells were defined as B-cells (CD3^−^, B220^+^), CD4/8-T-cells (CD3^+^, CD4^+^/CD8^+^), Th2-cells (CD3^+^, CD4^+^, GATA3^+^) and basophils (FcεR1α^+^). ILC2s were defined as Lin^−^, CD127^+^, and GATA3^+^ or ST2^+^ICOS^+^. Epithelial cells were defined with CD24 and Epcam in the CD45^−^ fraction. Gating strategies are shown in Supplementary Figures S4 and **Figure 4C**.

For intracellular (IL-5, IL-13) and nuclear staining (GATA3), cells were processed following cell surface staining using the Foxp3/Transcription Factor Kit (eBioscience). For intracellular cytokine detection, cells were cultured for 4 h in the presence of 1x cell stimulation and protein transport inhibitor cocktail (eBioscience) in RPMI-1640 culture media supplemented with 10 mM HEPES, 10% FCS, 100 mM 2-mercaptoethanol, 100 mg/ml penicillin, 100 IU/ml streptomycin. Flow cytometry analysis was performed as described above.

### RNA-Sequencing

Cells from stimulation experiments were harvested by centrifugation, transferred to Trizol (Life Technologies) and stored at −80°C until further processing. RNA was extracted from the aqueous trizol/chloroform phase using the RNeasy Mini kit (Qiagen) followed by off-column DNA digestion using Turbo DNase (Ambion). DNA-free RNA was cleaned and concentrated using an RNeasy Micro Kit (Qiagen) and assessed using a Bioanalyser (Agilent). RNA was processed for RNA sequencing using Ovation RNA-seq System V2 (Nugen), fragmented using the Covaris M220 and barcoded using Ovation Ultralow Library Systems (Nugen). Barcoded libraries were quantified using Qubit dsDNA BR assay (Invitrogen) and qPCR. Samples were sequenced using an Illumina Hiseq4000 (CRUK Cambridge Institute), and sequence data were aligned using Tophat2 with Partek Flow. RNA-seq analysis was performed using Partek® Genomics Suite® software, version 6.16 Copyright ©; 2016.

### LPS-Induced Lung Inflammation

Mice were treated intraperitoneally with either vehicle (10% kleptose) or iBET151 (10, 5, or 1 mg/kg) and after 1 h challenged intranasally with LPS (10 μg in 40 μl PBS) or PBS alone. Mice were weighed regularly before being sacrificed 48 h after LPS administration.

### IL-33-Induced Type-2 Lung Inflammation

Mice were treated intraperitoneally with either vehicle (10% kleptose) or iBET151 (10, 5, or 1 mg/kg) and challenged intranasally with IL-33 (0.2 μg in 40 μl PBS) on days 0 and 3 or PBS alone. All mice were sacrificed on day 6.

### Papain-Induced Lung Inflammation

Mice were treated with two intranasal doses of papain (25 μg on day 0 and day 1) or PBS in the presence or absence of iBET151 (10 mg/kg) or kleptose vehicle given daily for six days intraperitoneally.

### Ragweed-Induced Lung Inflammation

For the acute ragweed model, mice received either PBS alone or 100–300 μg of short ragweed (*Ambrosia artemisiifolia*) pollen extract (Greer Laboratories) intranasally, daily for 5 days, with or without iBET151 (10 mg/kg) treatment, and samples were taken on day 6. For the chronic lung inflammation model, mice received RWP, as above, administered intranasally three times per week over a 5-week period, and were sacrificed on day 38. iBET151 (10 mg/kg) or vehicle was given intraperitoneally for the final 10 days of the model.

### Plethysmography

Twenty-four hours after the final antigen challenge airways resistance was assessed using a restrained whole-body plethysmograph (EMMS, Hants, United Kingdom) as described previously (26). All baseline readings were found to be similar between experimental groups, and thus average pulmonary resistance after drug treatment was then divided by the baseline average for each mouse to determine fold induction. eDaq software was used to analyze airways resistance.

### Cytokine Analysis

Supernatants were harvested 1 day after IL-33 restimulation for cytokine analysis by Magpix cytometric bead array according to the manufacturer's instructions. IL-6 in bronchoalveolar lavage fluid was determined using the mouse IL-6 Ready-SET-Go! ELISA kit according to the manufacturer's instructions.

### Histology

Lung lobes were fixed in 10% formaldehyde in PBS overnight, dehydrated in 70% ethanol, and stored in PBS at 4°C until analysis. The CRUK Cambridge Institute Histology Core performed tissue embedding, sectioning and staining.

### Statistical Analysis

Graphpad Prism software was used for all statistical analysis. Significance levels are expressed as ^*^ <0.5, ^**^ <0.1, ^***^ <0.01 and ^****^ <0.001. RNA-seq analysis was performed using Partek® Genomics Suite® software, version 6.16 Copyright ©; 2016. The threshold for statistical significance for the *ex vivo* stimulated ILC2 cultures was set at a differential gene expression of >2-fold (unadjusted *p*-value). The threshold for differential gene expression for the *in vitro* cultured ILC2 assays was based on *Il13* expression (decreased by 1.7 fold), since IL-13 was shown by intracellular cytokine analysis and Magpix, to be decreased by iBET151 in the *in vitro* assays described above. Therefore, the threshold statistical significance was set at a differential gene expression of >1.7-fold (unadjusted p-value).

## Results

### iBET151 Inhibits Human ILC2 Activation

**Figure 1:**
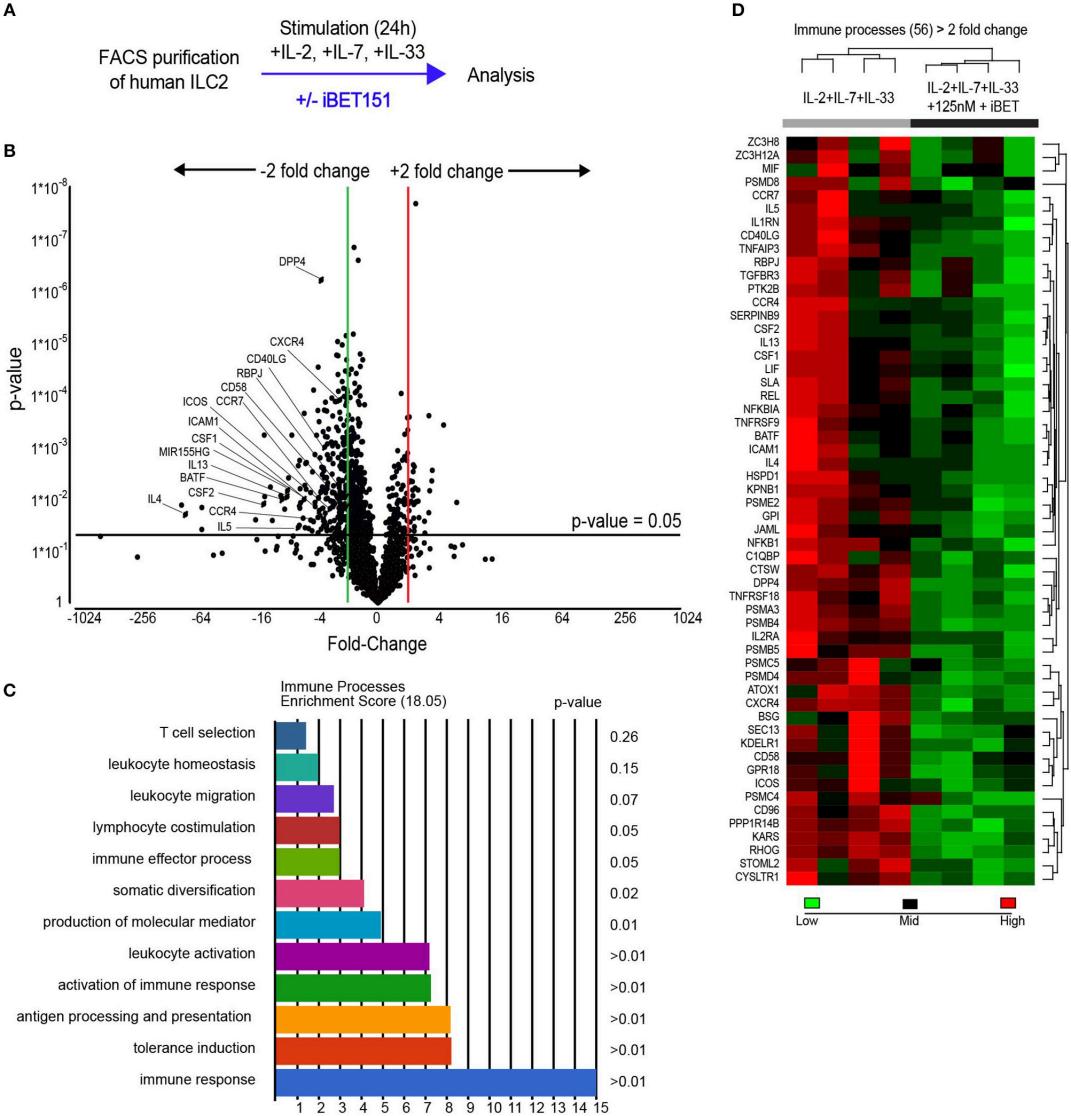
RNAseq analysis of iBET151-modulated genes expressed by *ex vivo* human ILC2s. **(A)** Method for stimulating hILC2s *ex vivo* with IL-33 and iBET151. **(B)** Volcano plot of genes differentially expressed between hILC2s stimulated with IL-33 alone or with IL-33 and 125 nM iBET151 (>2.0 fold change). **(C)** Gene ontology summary of immune system process genes lowered by iBET151. **(D)** Heat map of immune system process genes from gene ontology analysis in **(C)**. Data from four individual donors processed independently before library preparation.

To evaluate the potential of iBET151 to modulate human (h)ILC2 function we purified CD45^+^Lin^−^(CD8a, CD11b, CD11c, FcεRIα, CD123, CD20, CD56, CD71)CD127^+^CRTH2^+^ ILC2s from the peripheral blood of healthy donors (Supplementary Figure S1). To replicate the initial interaction with an epithelium-derived alarmin, purified ILC2s were cultured immediately with IL-2, IL-7, and IL-33 in the presence or absence of iBET151 for 24 h and then subject to RNA sequencing (iBET *ex vivo* ILC2s, Figure 1A). This analysis identified 270 statistically significant differentially expressed genes (>2.0-fold, unadjusted *p*-value). Within this subset, iBET treatment resulted in lower expression of 236 genes and gene ontology (GO) analysis indicated a further subset of 56 genes were involved in immune system processes (Figures 1B–D and Supplementary Figure S2A). Seven of the 34 genes, which were higher in iBET treated samples were also immune-related (Supplementary Figure S2B).

A characteristic feature of ILC2s is their copious expression of type-2 effector cytokines. Notably, treatment of ILC2s with iBET151 potently inhibited the transcription of the genes encoding IL-4, IL-5, and IL-13, as well as GM-CSF and CSF1 (Figures 1B,D). In addition, a number of cell surface receptors that are known to regulate ILC2 function were also decreased by iBET151. These included ICOS, CD40LG (CD154), ICAM1, and also CD58, a ligand for CD2, CD26 (DPP4), and receptors for lipid mediators from the eicosanoid pathway, for example CYSLTR1 (Figures 1B,D). ILC2 expression of the chemokine receptors CCR4, CXCR4, CCR7 that are known regulators of lymphocyte migration were also lower following iBET treatment (Figures 1B,D). The screen also identified the inhibition of RBPJ, a critical transcription factor in Notch signaling and ILC2 differentiation; BATF, a component of the BATF–AP1–IRF4 complex that regulates the type-2 cytokine locus in Th2 cells; and microRNA-155 host gene (HG) that is processed to give miR-155, which has been reported to support mouse ILC2 function (Figures 1B,D).

**Figure 2:**
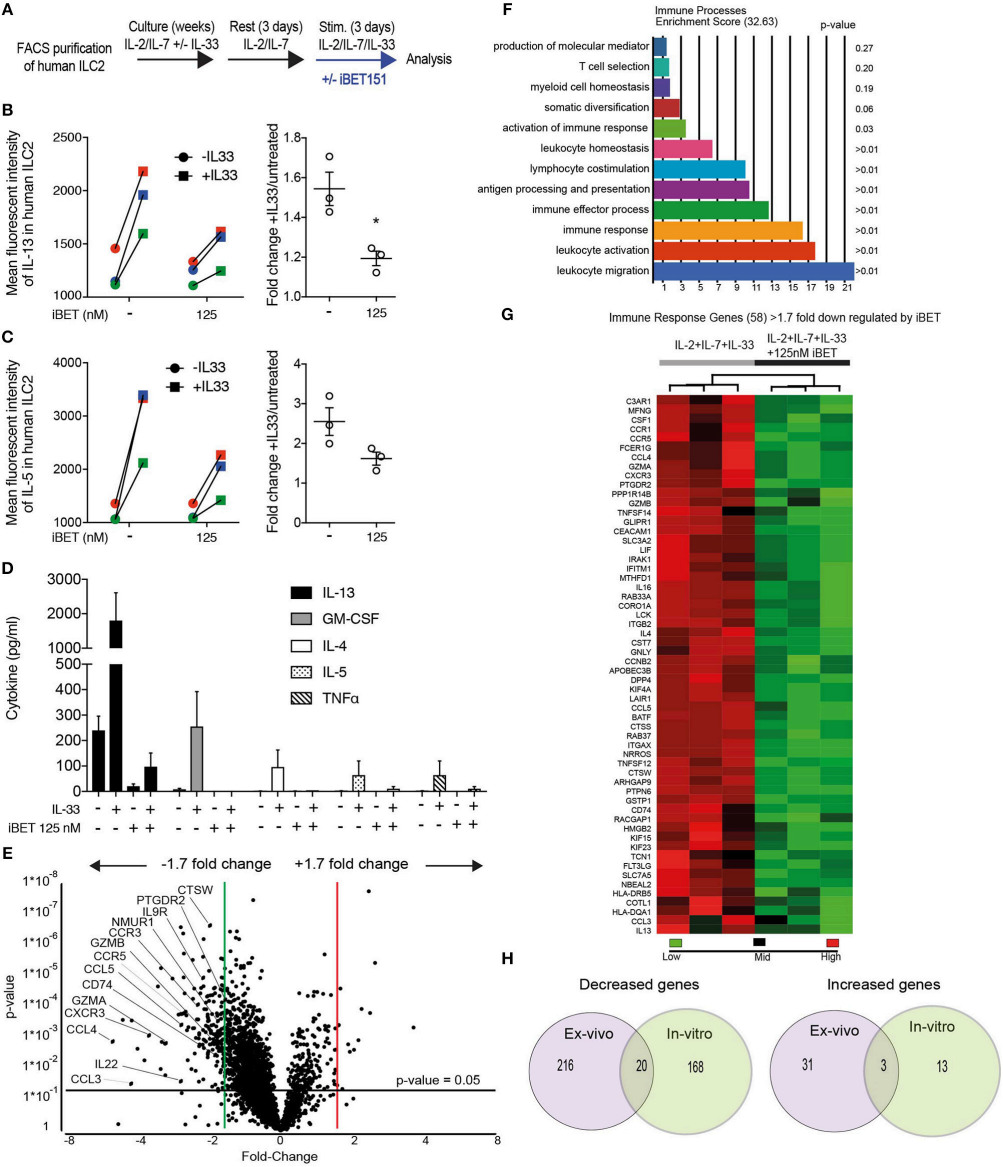
Analysis of iBET151-modulated proteins and genes expressed by *in vitro*-cultured human ILC2s. **(A)** Method for stimulating hILC2s *in vitro* with IL-33 and iBET151. Intracellular flow cytometry detection of **(B)**, IL-13 and **(C)**, IL-5 protein expression in hILC2 cultures in the absence or presence of iBET151. **(D)** Cytokine analysis of culture supernatant taken from *in vitro* expanded hILC2s following IL-33 restimulation for 24 h. **(E)** Volcano plot of genes differentially expressed between hILC2s stimulated with IL-33 alone or with IL-33 and 125 nM iBET151 (>1.7 fold change). **(F)** Gene ontology summary of immune system process genes lowered by iBET151. **(G)** Heat map of immune system process genes from gene ontology analysis in **(F)**. Data include three individual donors, processed independently before restimulation and library preparation. **(H)** Venn diagram of common and distinct genes from Figure 1C and Figure 2F.

To mimic chronic and highly activated type-2 inflammation, purified hILC2s were cultured in media containing IL-2 and IL-7 with IL-33 added to the cultures in cycles every alternate week (Figure 2A). After a period of expansion, the ILC2s were rested for 3 days in IL-2 and IL-7-containing media before being stimulated with IL-33 in the presence or absence of 125 nM iBET151 for 24 h. After 24 h, intracellular cytokine analysis was performed to detect the type-2 cytokines IL-13 and IL-5 in ILC2s. Inclusion of iBET151 in the cultures inhibited the production of IL-13 (Figure 2B), and to a lesser extent IL-5, following IL-33 stimulation (Figure 2C). Furthermore, analysis of culture supernatants indicated that type-2 cytokines (IL-4, IL-5, and IL-13) were inhibited by the presence of iBET151, as was GM-CSF (Figure 2D). Gene expression analysis using RNAseq was performed on the cultured ILC2s. In order to focus our analysis on functionally relevant genes we used the change in *Il13* expression (1.7 fold decreased) to set our threshold for genes that were lower following iBET treatment. This was based on intracellular cytokine analysis and Magpix, showing IL-13 to be decreased by iBET151 in the *in vitro* assays described above. Therefore, the threshold statistical significance was set at a differential gene expression of >1.7-fold (unadjusted *p*-value). We identified 204 genes with statistically significant differential expression between ILC2s stimulated in the presence or absence of iBET (Figure 2E and Supplementary Figure S3A). Within this subset, iBET treatment was found to lower the expression of 188 genes and GO analysis indicated 58 genes were involved in immune system processes (Figures 2F,G and Supplementary Figures S3A,B). A further subset of four of the 16 genes that showed iBET-dependent increases were immune system-associated (Supplementary Figures S3A,C).

Although several of the genes that were reduced by iBET151 in the long-term cultured ILC2s were in common with those from the 24-h *ex vivo* ILC2 cultures, there were a number of interesting additional targets (Figure 2H). These included Neuromedin U receptor (NMUR1, though at relatively low amounts), IL-9R and CRTH2 (prostaglandin D_2_ receptor); antigen processing and presentation genes (proteasome complex genes, CD74); chemokines and chemokine receptors (CCL3 (MIP1α), CCL4 (MIP1β), CCL5, CCR3, CCR5, CXCR3) (Figure 2E and Supplementary Figure S3). Interestingly, and mirroring recent reports on ILC plasticity, we observed increases in the transcription of NK/ILC3-like molecules in the long-term cultures. For example, Cathepsin (CTSW), Granzymes A and B (GZMA/B), and IL-22 (though at relatively low amounts) were increased relative to the *ex vivo* ILC2s. These molecules were inhibited by iBET151 treatment.

Taken together these results demonstrate that iBET151 is a potent inhibitor of type-2 responses in ILC2s, capable of repressing the expression of the signature type-2 cytokines, receptor-mediated ILC2 activation, and chemokine-regulated cell migration pathways.

### iBET151 Prevents LPS-Induced Lung Inflammation

**Figure 3:**
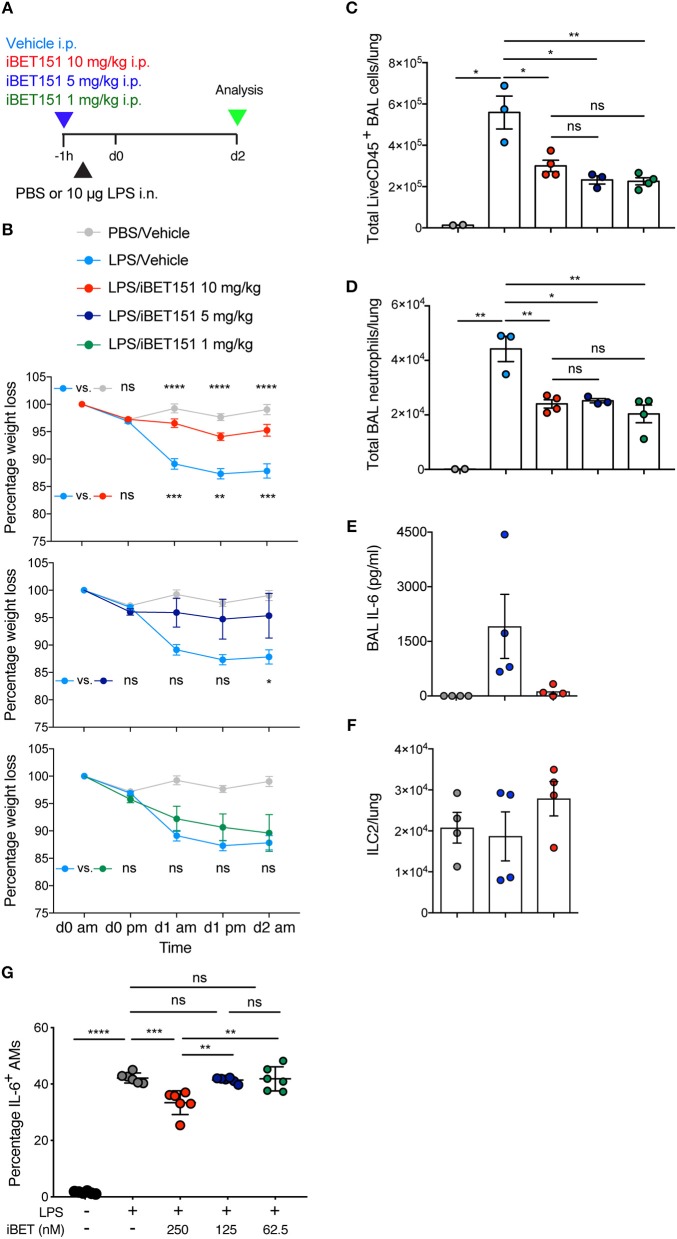
iBET-151 inhibits LPS-induced lung inflammation. **(A)** Model of LPS intranasal administration used to investigate the effects of iBET151. **(B)** Percentage weight loss, **(C)** total live CD45^+^ BAL cells, **(D)** total BAL neutrophils (liveCD45^+^SiglecF^−^Ly6G^+^F4/80^−^) of mice following treatment with LPS and vehicle (light blue circles), or LPS and iBET151 at 10 mg/kg (red circles), 5 mg/kg (dark blue circles), or 1 mg/kg (green circles). **(E)** IL-6 production in BAL fluid and **(F)**, total numbers of lung ILC2s following LPS administration and iBET151 treatment at 10 mg/kg. iBET151 10 mg/kg data are pooled from three repeat experiments, with iBET151 titration data being representative of one additional experiment. **(G)** Percentage of IL-6^+^ alveolar macrophages (AMs) following stimulation with LPS (100 ng/ml) and either 250, 125, or 62.5 nM iBET151 for 24 h.

BET inhibitors have been reported previously to be beneficial in type-1 inflammatory disease models (22, 24). To assess the ability of iBET151 to inhibit type-1 responses in the lung we administered LPS intranasally to mice to induce airways inflammation 1 h after intraperitoneal administration of iBET151 (Figure 3A). As expected, LPS treatment resulted in pronounced (~15%) weight loss within 2 days as compared to controls (Figure 3B). However, mice pre-treated with 10 mg/kg iBET151 were protected from LPS-induced weight loss (Figure 3B). This protective effect was maintained when iBET151 was given at 5 mg/kg, but the effect was lost at a dose of 1 mg/kg. iBET treatment at all doses (10, 5, and 1 mg/kg) was also associated with reduced infiltration of CD45^+^ cells into the bronchoalveolar lavage, primarily attributable to a fall in neutrophil (Ly6G^+^CD11b^+^) numbers (Figures 3C,D). Treatment of mice with 10 mg/kg iBET151 also reduced production of the pro-inflammatory cytokine IL-6 (Figure 3E). However, ILC2s (Figure 3F), CD103^+^ DCs, eosinophils (SiglecF^+^CD11c^−^) and alveolar macrophages (SiglecF^+^CD11c^+^) were not altered (data not shown). Furthermore, iBET151 treatment of *in vitro* cultured LPS-stimulated alveolar macrophages resulted in a significant decrease in IL-6 production at 250 nM of iBET151, but not at 125 or 62.5 nM (Figure 3G). Taken together these results indicate that iBET151 protected mice from LPS-induced weight loss, inflammatory cell infiltration and inflammatory cytokine production.

### iBET151 Restrains Type-2 Lung Inflammation in a Papain Challenge Model

**Figure 4:**
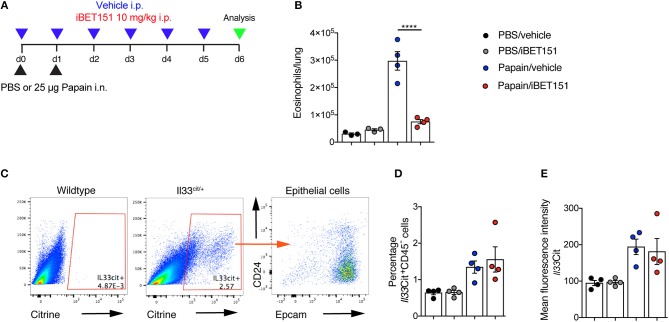
iBET inhibits papain-induced eosinophilia in the lung in an IL-33-independent manner. **(A)** Model of papain-induced lung inflammation used to investigate the effects of iBET151. **(B)** Total number of eosinophils in the lung following treatment with papain and vehicle (blue circles) or papain and iBET151 at 10 mg/kg (red circles). **(C)** Gating strategy for the identification of *Il33*-citrine^+^ lung epithelial cells, **(D)** percentage, and **(E)** mean fluorescence intensity of *Il33*-citrine^+^ cells in the lungs following intranasal papain challenge in the presence or absence of iBET-151. Data are representative of two repeat experiments.

Papain is a cysteine protease that when administered intranasally to mice leads to lung damage and results in type-2 inflammation. This has been shown to be highly dependent on IL-33 (27). Mice were treated with intranasal papain in the presence or absence of iBET151 and lung inflammation was assessed 5 days after the final administration of papain (Figure 4A). At this timepoint iBET151 inhibited eosinophil accumulation in the lungs (Figure 4B), but we could not detect changes in ILC2 or T cell numbers (data not shown). We speculated whether this result may be due to iBET151 blocking the expression of *Il33* by the epithelial cells, thereby curtailing the response at initiation, or by directly altering the cell-intrinsic properties of the inflammatory cells. To address this question, we used *Il33*-citrine (*Il33*^*cit*/+^) reporter mice in which the citrine fluorescent protein is expressed from the start site of the *Il33* gene providing a surrogate marker for *Il33* transcription. *Il33*-citrine mice were challenged intranasally with papain in the presence or absence of iBET151 and the proportions of *Il33*-citrine-positive cells determined after 6 days. Although papain induced *Il33-*citrine expression in CD45^−^CD24^−^Epcam+ epithelial cells this was unaltered by co-treatment of the mice with iBET151 (Figures 4C–E). These data indicate that whilst iBET151 can inhibit type-2 lung inflammation in a papain-induced model this is not due to altered transcription of the *Il33* locus in epithelial cells.

### iBET151 Inhibits IL-33-Induced Type-2 Lung Inflammation

**Figure 5:**
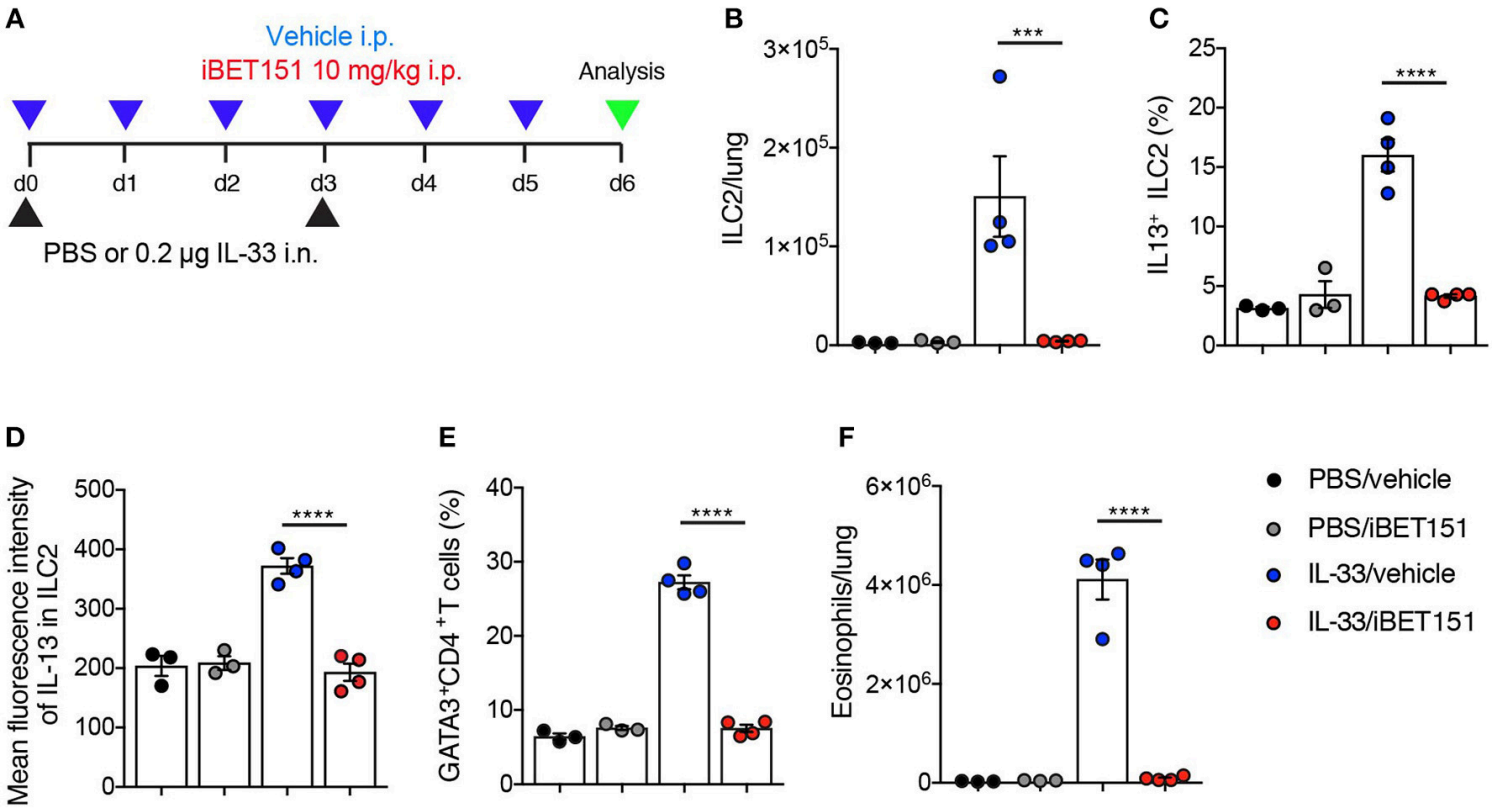
iBET inhibits IL-33-induced type-2 inflammation in the lungs. **(A)** Model of IL-33-induced lung inflammation used to investigate the effects of iBET151. **(B)** Total number of lung ILC2s, **(C)** percentage of IL-13^+^ ILC2s, **(D)** mean fluorescence intensity of IL-13 expression by ILC2s, **(E)** percentage of lung Th2 cell number (GATA3^+^CD4^+^) and **(F)** total number of eosinophils in the lung following IL-33 intranasal challenge and vehicle (blue circles), or IL-33 and iBET151 at 10 mg/kg (red circles). Data are representative of three repeat experiments.

IL-33 is an epithelium-derived “alarmin” cytokine that potently induces type-2 immune reactions. To determine if the iBET151 suppression of allergen-induced type 2 lung inflammation was up- or down-stream of IL-33, we took the approach of treating mice with IL-33 in the presence or absence of iBET151 at 10 mg/kg (Figure 5A). Intranasal administration of IL-33, with intraperitoneal treatment of vehicle, resulted in an increase of IL-13-producing ILC2s (Figures 5B–D), GATA3^+^CD4^+^ Th2 cells (Figure 5E), and eosinophils (Figure 5F) by day 6 post-treatment. Notably, daily administration of iBET151 completely prevented all facets of IL-33-induced type-2 immunity measured, including inhibiting ILC2 numbers and their expression of IL-13, blocking Th2 cells and preventing lung eosinophilia (Figures 5B–F). Titration of iBET151 to 5 mg/kg, but not 1 mg/kg, also caused significant decreases in type 2 inflammation (Supplementary Figure S5). However, titration of iBET151 to a dose of 1 mg/kg still resulted in a statistically significant reduction in lung ILC2 percentage (Supplementary Figure S5A). Furthermore, *in vitro* culture of IL-33-induced ILC2s with decreasing doses of iBET151 (250, 125, and 62.5 nM) also showed a dose dependent effect on IL-13 production (Supplementary Figure S5E). However, iBET151 showed no ability to effect changes in ILC2 viability (live/dead staining) or proliferation (Ki67 expression, data not shown). Thus, iBET151 is a potent inhibitor of IL-33-induced inflammation *in vivo*.

### iBET151 Curbs Type-2 Responses in RWP-Induced Allergic Lung Inflammation

**Figure 6:**
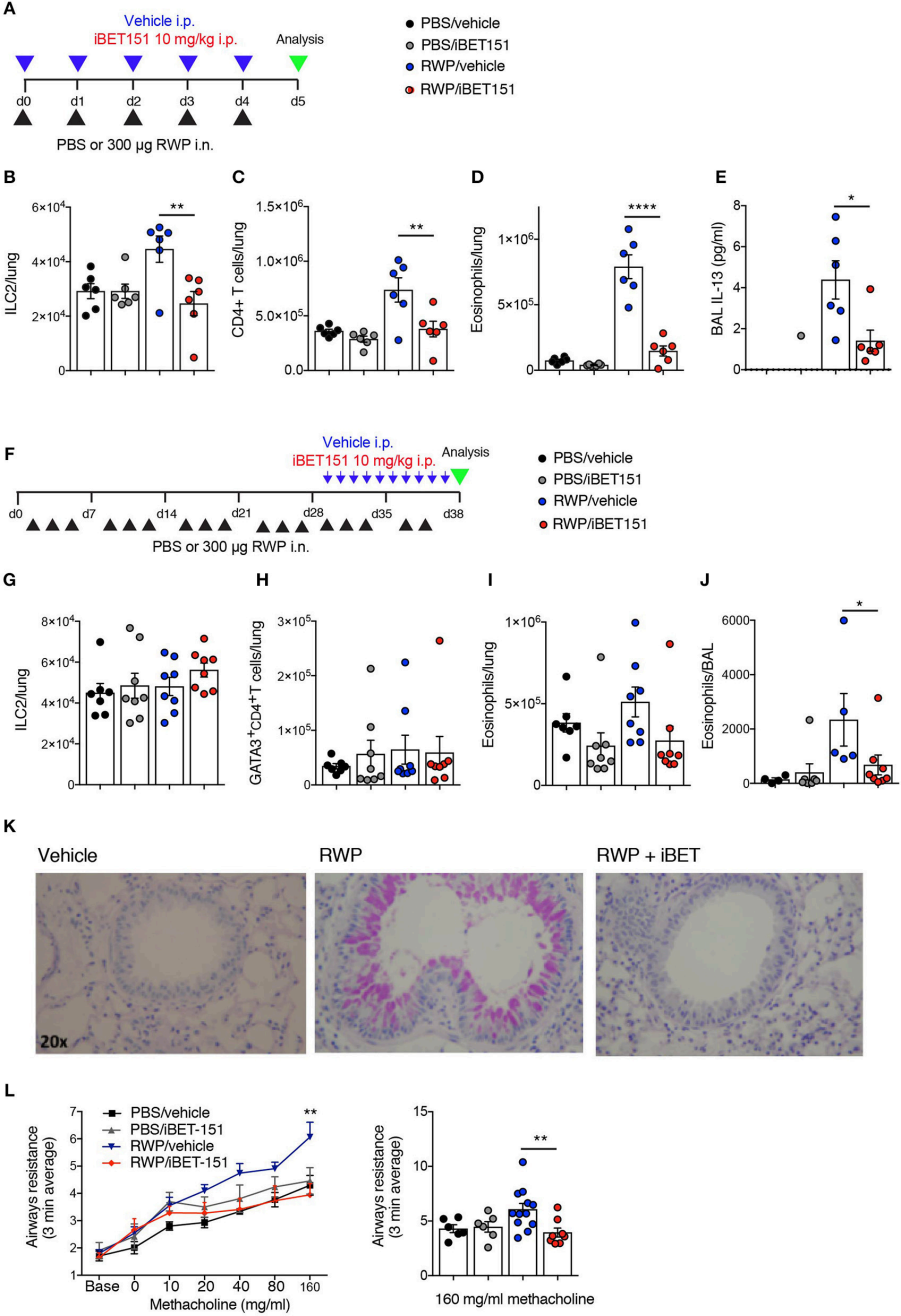
iBET reduces type-2 allergic inflammation in acute and chronic ragweed protein (RWP) challenge models. **(A)** Model of RWP-induced acute lung inflammation used to investigate the effects of iBET151. **(B–D)** Total number of immune cells in the lung, and **(E)** IL-13 in the BAL fluid of mice challenged with intranasal RWP for 5 consecutive days in the presence or absence of iBET-151. **(F)** Model of RWP-induced chronic lung inflammation used to investigate the effects of iBET151. Total number of immune cells **(G–I)** in the lung, and **(J)** in the BAL fluid of mice challenged with intranasal RWP three times per week over a 5-week period in the presence or absence of iBET-151 given intraperitoneally for the final 10 days of the model. **(K)** Lung histology and **(L)** airways resistance measurements from mice described in **(G–J)**. Data are representative of two repeat experiments.

Next, we wished to assess the ability of iBET151 to modulate type-2 immunity induced in response to a clinically relevant allergen (Figure 6A). Ragweed pollen (RWP) is one of the most common causes of allergies, especially in the United States. As expected, RWP challenge over 6 days resulted in increased numbers of ILC2s (Figure 6B), CD4^+^ T cells (Figure 6C) and eosinophils (Figure 6D) in the lungs, and IL-13 (Figure 6E) in the BAL. Notably, co-administration of iBET151 completely inhibited this inflammatory response with lung ILC2s, CD4^+^ T cells, eosinophils and BAL IL-13 remaining at baseline (Figures 6B–E). Thus, iBET151 has a potent inhibitory effect on acute type-2 inflammation in the lungs.

We have previously established a prolonged lung inflammation model in which RWP is administered intranasally over a 5-week period (Figure 6F). Following RWP challenge, flow cytometry and lung histology confirmed the existence of lung pathology, characterized by eosinophilia and goblet cell hyperplasia (Figures 6I–K). When iBET151 was given intraperitoneally for the final 10 days of the model it inhibited allergen-induced eosinophilia and mucus production (Figures 6I–K). We were unable to detect changes in ILC2s or Th2 cell numbers in this model in the presence or absence of RWP or iBET151 (Figures 6G,H). However, increases in methacholine-induced airways hyperreactivity in RWP challenged mice were blocked by daily administration of iBET151 during the final 10 days of allergen challenge (Figures 6L).

Together, these data demonstrate that iBET151 is a potent inhibitor of both acute and established type-2 immune mediated lung inflammation.

## Discussion

We have assessed the effects of the bromodomain inhibitor iBET151 on human ILC2 gene expression in response to activation in the presence of IL-33. Gene expression analysis using RNA-sequencing identified that iBET151 was a potent inhibitor of several immune regulatory molecules that have been shown to be important for ILC2-mediated immune activation. Critically, this included the type-2 effector cytokines IL-4, IL-5, and IL-13, as well as GM-CSF and CSF1. The roles for IL-4, IL-5, IL-13, and GM-CSF in allergic diseases are well established (28), and it is clear that iBET151 has the potential to suppress these responses in ILC2. Although there is currently no report of a role for CSF1 relating to human or mouse ILC2s, airway epithelial cell-derived CSF1 was reported recently to activate cDC2 and promote allergen sensitization (29).

To complement the *in vitro* analysis of human ILC2 we performed additional studies to assess the efficacy of iBET151 in inhibiting type-2 immune-mediated inflammation in mouse lungs. Strikingly, iBET151 completely blocked all IL-33-induced responses in the lungs following intranasal challenge. iBET151 treatment also successfully prevented type-2 responses induced by more complex allergens, including papain and RWP extract. To determine if iBET151 was acting directly on the epithelial cells to preclude the transcriptional activation of the *Il33* gene we examined the expression of citrine in epithelial cells derived from *Il33*-citrine reporter mice. Interestingly, citrine levels were not altered by iBET151 treatment, suggesting that iBET151 inhibition of the type-2 response acts downstream of *Il33* gene expression by altering gene expression in IL-33-responsive cells such as ILC2. As shown previously, and herein, ILC2 are key IL-33-responsive cells that help drive IL-5 and IL-13 levels in acute type-2 responses. Indeed, in the case of the type-2 response to RWP, administration of iBET151 correlated with fewer ILC2 and fewer CD4^+^ T cells. Furthermore, in a long-term model of lung inflammation induced by RWP, iBET151 treatment administered over only the last 10 days of the allergen challenge model inhibited mucus production, eosinophil infiltration and airway hyperreactivity, though we could not detect changes in ILC2s or T cells. Taken together these data demonstrate that iBET151 has potent inhibitory effects on ILC2s in type-2 immunity and successfully counteracts lung inflammation. Also, the results may suggest alternative ILC2- and T cell-proliferation independent pathways for iBET151 inhibition of chronic eosinophilia and mucus production. This could perhaps be explained by a direct effect of iBET151 on the epithelium (30), or by changes in type-2 cytokine production by ILC2s and T cells, though this was not measured in our studies.

In addition to effector cytokines, iBET151 inhibited the expression of numerous other genes in ILC2s that may contribute to the efficacy observed in *in vivo* models. This includes a number of cell surface receptors with known roles as ILC2 or lymphocyte activators, including ICOS, CD40L and ICAM1. ICOS expression was first reported on mouse ILC2 (4, 31), and ICOS:ICOS ligand (ICOSL) signaling has since been shown to be important for ILC2 function, including their role in the induction of airway hyperreactivity (7). Additionally, recent evidence indicates that induced Tregs (iTregs) can suppress the production of ILC2-derived IL-5 and IL-13 via the ICOS-ICOSL pathway (32, 33). CD40 ligand was reported recently to be induced on ILC2s in response to stimulation with phorbol ester and ionomycin, IL-25 or IL-33, or a number of TLR agonists. Notably, following the induction of CD40L, these ILC2s induced immunoglobulin production, including IgE, by B cells (34). Furthermore, ICAM1-deficient ILC2s were reported to produce diminished levels of type-2 cytokines in response to IL-33 stimulation, and ICAM1-deficient mice developed less severe airway inflammation following lung challenge with *Alternaria alternata* fungal extract or papain (6).

Although there are currently no reports of roles for CD58, CD26/DPP4, or CD96 in human or mouse ILC2-mediated responses, these molecules are expressed on ILC2 and inhibited by iBET151. CD58 is a ligand for CD2 and antibodies binding to CD2 resulted in increased T cell production of IL-4 (35), suggesting that modulating CD58 expression on ILC2s may have consequences for interactions between ILC2 and Th2 cell and subsequently for cytokine production. Interestingly, CD26 (DPP4) is a dipeptidyl peptidase-4 known to play roles in T cell homeostasis and activation, including the interaction of APCs with T cells. Many neuropeptides, hormones, growth factors, and chemokines have the X-Pro or -Ala motif at their N-terminus and have been shown to be cleaved by DPP4, leading to their enhanced degradation by further peptidases. In addition, DPP4 has also been reported to bind several cell surface receptors (e.g., Caveolin on APCs) to regulate various physiological and immune responses. Although soluble CD26/DPP4 has been reported to increase in asthmatics, its contribution is still contentious (36).

In contrast to the extensive number of activation molecules that were inhibited by iBET151 we also noted that CD96, an inhibitory receptor for CD155 was also lower (37). CD96 is expressed by CD4^+^ and CD8+ T cells, and NK cells in humans and mice and is a member of the nectin-like protein family. The absence of CD96-signaling in mice resulted in improved resistance to melanoma metastasis (38), and it will be interesting to discover how expression of this molecule specifically on ILC2s may regulate immunity.

We also identified that in ILC2s iBET151 repressed the expression of pathways that are known to regulate lymphocyte migration, including the chemokine receptors CCR4, CXCR4, and CCR7. CCR4 has been associated with asthma (39), and CCR4-deficient mice display reduced airway hyperresponsiveness following chronic lung allergen challenge (40). Furthermore, a CCR4 antagonist attenuated allergic lung inflammation in an experimental mouse model (41). Although impaired migration of ILC2s may be related to these observations, an effect on CCR4^+^ Th2 migration cannot be excluded. CXCR4 binds stromal-derived-factor-1 (SDF-1 also called CXCL12), a potent chemotactic molecule for lymphocytes, and a CXCR4 inhibitor has been reported to attenuate allergen-induced lung inflammation (42). CCR7 is also involved in homing of T cells to secondary lymphoid tissues including the lymph nodes and spleen. It is expressed on mouse ILC1 and ILC3, but only at low levels on ILC2 (43). The potential roles of these chemokines in human ILC2 function requires further investigation.

The expression of bioactive lipid products by ILC2 is also inhibited by iBET151. Arachidonic acid metabolism leads to the production of either prostaglandins via the cyclooxygenase (COX) pathway, or leukotrienes through the 5-lipooxygenase (5LO) pathway (44). Our data indicate that ILC2 expression of the receptor for cysteinyl leukotrienes, CYSLTR1, was lowered by iBET151. CysLTs have been reported to potentiate type-2 cytokine production from ILC2s stimulated with IL-33 and CYSLTR-deficient mice display reduced ILC2 responses following exposure to *A. alternata* allergen (45).

A number of notable transcription factors were also reduced by treatment of ILC2 with iBET151, including RBPJ, BATF, and EGR2. RBPJ is a transcriptional regulator critical for Notch signaling and has been shown to be essential for mouse and human ILC2 development (31, 46) and plasticity (47). BATF–AP1–IRF4 complexes regulate the type-2 cytokine locus in Th2 cells to induce IL-4 that feeds back to induce Th2 cell differentiation and amplify the subsequent Th2 cell immune response (48–52). Recently, BACH2 has been shown to antagonize this feedback loop by disrupting BATF–AP1–IRF4 complex binding to AICE motifs in the type 2 cytokine locus (50). It has been reported that BATF transcription is significantly higher in the PBMC of children with recurrent asthma who have not been treated with corticosteroid (53). In addition, Batf-deficient mice were less susceptible to experimental allergic lung inflammation. Though there is currently no report of a role for BATF in human or mouse ILC2s it is highly likely that this pathway will be important for ILC2-derived type-2 cytokine production. The transcription factor EGR2 (early growth response 2) suppresses excessive immune activation and Egr2-deficient T cells are hyperproliferative (54). There is currently no report of a role for EGR2 in human or mouse ILC2s. Furthermore, a recent report demonstrates that in cat dander extract-induced lung inflammation, bromodomain-containing protein 4 (Brd4) and transcription factor NF-κB/RELA interaction is crucial. Inhibition of Brd4 confers protection by inhibiting IKK-NF-κB/RELA mediated epithelial to mesenchymal transition and mucosal inflammation (30). In our study, iBET151 treatment shows downregulation of NFKB1 gene in human ILCs indicating the involvement of common pathways in immune and non-immune cells.

Interestingly, we also found that miR-155HG expression was lowered in ILC2 in response to iBET151. MiR-155HG is processed to give miR-155, and inhibitors of miR-155 have been reported to decrease IL-13 and IL-5 expression by Th2 cells, and miR-155-deficient mice exhibited reduced airway inflammation and eosinophilia in response to house dust mite challenge or a model of ovalbumin-induced allergic airway inflammation (55, 56). Recently, miR-155 has also been reported to regulate ILC2 and it was observed that miR-155-deficient mice have reduced numbers of ILC2 in response to ovalbumin-induced lung inflammation (57, 58).

Following extended culture in the presence of IL-33 the ILC2s retained their propensity for type-2 cytokine expression, but also showed a higher expression of additional markers of allergic-type activation including iBET-sensitive CRTH2, IL-9 receptor and the receptor for the neuropeptide, Neuromedin U. CRTH2, encoded by the *PTGDR2* gene, is the receptor for prostaglandin D_2_ and is expressed by a spectrum of type-2 leukocytes, including Th2 cells, eosinophils and ILC2s. CRTH2 is a key marker for the identification of ILC2s (11) and its ligation regulates ILC2 migration and activation (18). Notably, fewer ILC2 accumulate in the lungs of CRTH2-deficient mice in response to pulmonary challenge (59). Inhibitors of the PGD_2_ pathway are in clinical trials for allergic diseases and it is likely that CRTH2 and PGD_2_ inhibitors will also inhibit ILC2 biology. IL-9R expression was increased in the long-term ILC2 cultures and was decreased by iBET151. IL-9 has been shown to act as an autocrine growth factor for ILC2 (60). Notably, we also identified that the gene encoding Neuromedin U receptor 1 (*NMUR1*) is induced following long-term ILC2 culture and is reduced by iBET151. Several groups have recently reported that Neuromedin U receptor expression on mouse ILC2s is critical for their activation by Neuromedin U produced by mucosal neurons. Without this signal helminth worms are expelled less efficiently (61–63). Our results suggest that human ILC2 also upregulate the Neuromedin U receptor and therefore may acquire responsiveness to neuron-derived signals.

Extended culture also led to the expression of additional chemokines and chemokine receptors that could be inhibited by co-culture with iBET151. The chemokines CCL3, CCL4, and CCL5 are all associated with the migration of macrophages and other leukocytes through their binding to CCR1, CCR4, and CCR5, and have been linked with asthma. Notably, CCR3 is the receptor for eotaxins that contribute to the accumulation and activation of eosinophils and T cells in the airways (64). Eotaxins are increased in asthma and correlate with disease severity. CCR3 also binds MCP-3 (CCL7), MCP-4 (CCL13), and RANTES (CCL5). There is currently no report of a role for CCR3 in human ILC2s, but it would be predicted that the expression of this receptor would facilitate ILC2 migration to sites of type-2 inflammation. Inhibitors of CCR3 are in clinical trials for asthma (65).

We have shown previously that mouse and human ILC2s are capable of processing and presenting antigens to T cells (8). RNAseq analysis identified that iBET151 treatment efficiently inhibited the transcription of ILC2-expressed genes encoding proteins involved in antigen processing and presentation. These included subunits of the proteasome complex that is required for enzymatically processing proteins into oligopeptides in the cytoplasm. In addition, CD74, the invariant chain of the MHCII complex, and HLA-DR and DQ were also inhibited. These results suggest that iBET151-treated ILC2 would be less efficient at processing and presenting antigens to T cells. Taken together, our results indicate iBET151 could be acting as an inhibitor of multiple transcriptional pathways responsible for mediating ILC2 migration, proliferation, and activation. Further studies would be required to delineate the epigenetic changes involved in these processes.

Our data suggest that iBET inhibitors could have therapeutic potential in allergic diseases and asthma. Based on their anti-tumor effects *in vitro* and in animal models, several BET bromodomain inhibitors have entered clinical trials in oncology. In addition to evaluating therapeutic efficacy in cancer, these trials are important for identifying potential side effects of BET inhibition in humans, which have included thrombocytopenia, diarrhea, and vomiting. We found that iBET151 was well-tolerated in mouse models of allergic lung inflammation and it is possible that treatment of inflammation in humans may require lower doses than have been administered in cancer studies and led to unwanted side-effects. At present this type of pan-iBET inhibitor would downmodulate multiple inflammatory networks, which could promote an increased susceptibility to microbial infections. However, such effects also arise when giving high dose oral steroids to severe asthmatics, and indeed perhaps pan-iBET inhibitors would offer an alternative to steroid use in such patients, and in cases of non-allergic or steroid resistant asthma. As inhibitors of individual BET bromodomain family members become available it will be important to determine if these may be used to preferentially modulate type-2 immunity. Their potential efficacy also raises the possibility of using BET inhibitors in combination therapies for allergic disease, possibly to synergise with anti-cytokine treatments.

## Ethics Statement

All mouse experiments undertaken in this study were performed with the approval of the UK Home Office. All experiments involving human subjects were performed according to the research ethics committee of NHS health and research authority guidelines, reference no. 15/LO/0321.

## Author Contributions

BK designed and performed experiments and wrote the paper. JB and HJ performed experiments, provided advice on experimental design. BR, MG, DJ, and AP analyzed the gene expression data. MB, DT, and AvO provided expertise, advice on experimental design and interpretation, and reagents. AM supervised the project, designed the experiments, and wrote the paper. All authors commented on the manuscript.

### Conflict of Interest Statement

AM has grant funding from GSK and AstraZeneca/MedImmune. MB, DJ, AP, DT, and AvO are employees of GSK. The remaining authors declare that the research was conducted in the absence of any commercial or financial relationships that could be construed as a potential conflict of interest.

## Supplementary Material

Supplementary Figure S1 Human blood ILC subsets analyzed by flow cytometry.

Supplementary Figure S2 RNAseq analysis of iBET151-modulated genes expressed by *ex vivo* human ILC2s. **(A)** List of the 270 differentially expressed genes between IL-33-stimulated hILC2 and IL-33-stimulated iBET (125 nM)-treated hILC2 from heatmap in Figure 1. **(B)** List of immune system process-associated genes increased by iBET151 following *ex vivo* culture of ILC2s.

Supplementary Figure S3 Gene ontology analysis of iBET151-modulated genes expressed by *in vitro*-cultured human ILC2s. **(A)** List of the 204 differentially expressed genes between IL-33-stimulated hILC2 and IL-33-stimulated iBET (125 nM)-treated hILC2 from heatmap in Figure 2. **(B)** List of immune system process-associated genes reduced by iBET151 following *in vitro* culture of ILC2s. **(C)** List of immune system process genes from gene ontology analysis increased by iBET151 following *in vitro* culture of ILC2s.

Supplementary Figure S4 Flow cytometry gating strategy for **(A)** mouse lymphoid cell lineages, and **(B)** myeloid cell lineage.

Supplementary Figure S5 Titration of iBET151 in IL-33-induced type-2 inflammation in the lungs. **(A)** Percentage of ILC2s, **(B)** percentage of IL-13^+^ ILC2s, **(C)** mean fluorescence intensity of IL-13 expression by ILC2s, and **(D)** percentage of eosinophils in the lung following IL-33 intranasal challenge and vehicle (light blue circles), or IL-33 and iBET151 at 10 mg/kg (red circles), 5 mg/kg (dark blue circles), or 1 mg/kg (green circles) (as per Figure 5A). **(E)** Percentage of IL-13^+^ ILC2s following stimulation *in vitro* with IL-7 and IL-33 and either 250, 125, or 62.5 nM iBET151 for 24 h. iBET151 titration data are from one experiment.
