# Supplementary material for: BET Bromodomain Inhibitor iBET151 Impedes Human ILC2 Activation and Prevents Experimental Allergic Lung Inflammation
Source: Front Immunol. 2019 Apr 9;10:678. doi: 10.3389/fimmu.2019.00678 (PMC6465521; doi:10.3389/fimmu.2019.00678)

Supplementary Figure S1

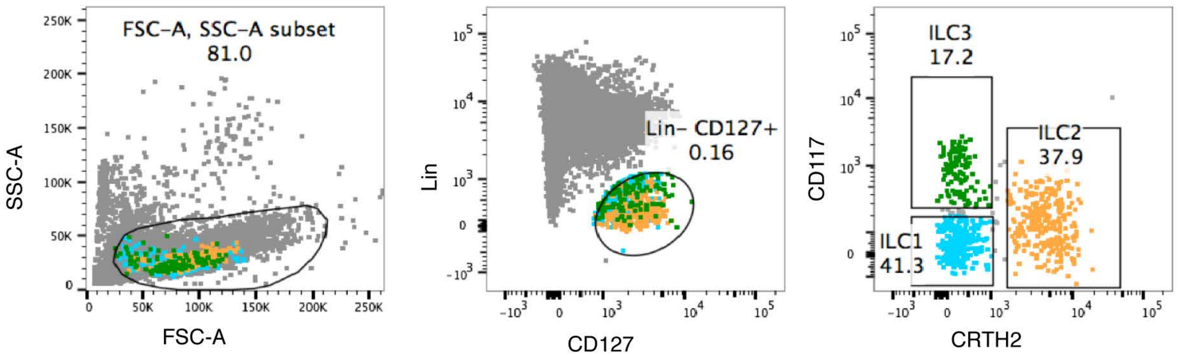

Supplementary Figure S2.

A

ARHGEF3
ASF1A
CDC25B
CLCC1
CSNK1D
CTDSP2
EGR1
EIF3F
GLIPR2
HEXIM1
HGSNAT
KANSL2
KHNYN
KIAA0040
KLF3
KLHDC2
LTB
LUC7L
MAF
MTERF1
NDRG1
NEAT1
NPIPB4
NTAN1
OSTF1
POLE4
PRKY
RABIF
SCARNA28
TCP11L2
THUMPD3-AS1
TRAT1
WDTC1
ZNF592
AATF
AHI1
AK2
ANP32B
ANP32E
ATIC
ATOX1
ATP5F1B
ATP5F1C
ATP6V1E1
B3GALT6
BATF
BCL2L1
BLMH
BRIX1
BSG
BUD31
C11orf98
C19orf53
C1QBP
C1orf43
CCDC59
CCR4
CCR7
CCT5
CCT6A
CD40LG
CD58
CD96
CHCHD1
CHCHD2
CLIC1
COA3
COA4
COMMD1
COX5B
COX6C
CPSF4
CRTC2
CSF1
CSF2
CSTF3
CTSW
CXCR4
CYB5B
CYSLTR1
CYTOR
DCTPP1
DDX10
DPP4
EBNA1BP2
ECD
EFHD2
EGR2
EIF2A
EIF4A1
EIF6
ETFA
FBL
FKBP3
FKBP4
FURIN
GNA15
GPI
GPR171
GPR18
GTPBP4
HAX1
HEATR1
HPGDS
HSD17B10
HSPA4
HSPA9
HSPD1
HYPK
IARS
ICAM1
ICOS
IL13
IL1RN
IL2RA
IL4
IL5
JAML
KARS
KDELR1
KPNB1
LAPTM4A
LDHA
LIF
LRRC59
MALSU1
MIF
MIR155HG
MIR3142HG
MMADHC
MPV17L2
MPZL3
MRPL20
MRPL24
MRPL27
MRPL36
MRPS15
MRPS24
MRPS7
MT2A
MTHFD2
MTIF2
MYC
NABP2
NAMPT
NARS
NASP
NDUFA12
NDUFA3
NDUFB9
NFKB1
NFKBIA
NFKBIZ
NFYC
NGLY1
NHP2
NINJ1
NME1
NOP58
NQO1
NRBF2
NSMCE1
NSUN2
OCIAD2
PAICS
PARK7
PDE4A
PDGFA
PHB2
PLAGL2
PLIN2
POLR2K
PPA1
PPID
PPP1R14B
PSD4
PSMA3
PSMB4
PSMB5
PSMC4
PSMC5
PSMD4
PSMD8
PSME2
PTGS2
PTK2B
PTPN7
PUF60
PUM3
RABGGTB
RAD50
RAN
RBBP8
RBPJ
RCC2
RCN1
REL
RGS16
RHBDD2
RHOG
RINT1
RIOK1
RNF19A
RPL35
RPL36
RPS10-NUDT3
RTL8A
SAMD4B
SART3
SCAMP3
SDHB
SEC13
SEC61B
SELENOF
SENP1
SERP1
SERPINB9
SF3B5
SGPL1
SH2D2A
SLA
SLC35B1
SLC35B2
SLC43A3
SLC50A1
SLIRP
SNHG8
SNRPB2
SNU13
SPCS1
SQOR
SRGN
SRM
SRPRB
STAT4
STAT5A
STOML2
TCP1
TCTN3
TFRC
TGFBR3
TIMM10
TKT
TMEM41A
TNFAIP3
TNFRSF18
TNFRSF9
TP53
TRAF1
TRMT10C
TRMU
TUBA4A
TUFM
TXNL1
UBAP1
UBE2F
UBE2L6
UCHL1
UFC1
UTP4
WARS
WDR43
XPO5
YARS
ZBED2
ZC3H12A
ZC3H8
ZFAND2A
ZMAT2
ZNF207

B Upregulated immune system genes

TRAT1
OSTF1
NDRG1
LTB
HGSNAT
HEXIM1
EGR1

Supplementary Figure S3

A

ACSL5	CCL5	CRELD2	GLIPR1	HIST3H2BB	LAIR1	NMUR1	RNVU1-19	TNFSF12
ADGRG1	CCNA2	CSF1	GNLY	HJURP	LCK	NOP16	RPS19BP1	TNFSF14
ANP32B	CCNB1	CST7	GOLGA8O	HLA-DQA1	LIF	NRROS	SAMD10	TOP2A
ANP32E	CCNB2	CTSS	GPATCH4	HLA-DRB5	LINC00892	NSMCE1	SASH3	TPX2
AOAH-IT1	CCR1	CTSW	GPR171	HLA-DRB6	LMNB1	NUDT1	SAT1	TSPOAP1-AS1
APOBEC3B	CCR3	CXCR3	GPR68	HMGB2	MALAT1	OCEL1	SDF2L1	TUBA1B
APOBR	CCR5	DCXR	GSTP1	HMMR	MBOAT7	PDCL3	37135	TUBA1C
APOL3	CD74	DDX39A	GTF3C6	HOPX	MCM2	PDE7B	SH2D2A	TUBA4A
ARHGAP11A	CDC20	DLGAP5	GTSE1	IDH2	MFNG	PHF19	SKA1	UBE2C
ARHGAP9	CDCA3	DPM3	GZMA	IFITM1	MICAL2	PLK1	SLC39A8	UFC1
ASB2	CDCA5	DPP4	GZMB	IL13	MIR142	PLPP1	SLC3A2	VAMP5
ATG16L1	CDCA8	EBP	HCST	IL16	MIR3064	PMCH	SLC7A5	ZWINT
AURKA	CDKN3	EGR1	HDGF	IL22	MKI67	PPIF	SNRPF	
BATF	CEACAM1	EGR2	HEXIM1	IL4	MPZL3	PPP1R14B	SPAG5	
BIRC3	CENPA	ELOVL6	HINT2	IL9R	MRPS24	PRC1	SSRP1	
BIRC5	CENPF	EMP3	HIRIP3	INCENP	MTHFD1	PRR11	STMN1	
C15orf53	CENPX	ESPL1	HIST1H2AL	IRAK1	MYBL2	PTGDR2	SUV39H1	
C3AR1	CEP55	FCER1G	HIST1H2BE	ITGAX	MZT2A	PTGIS	TARP	
CAPG	CKAP2L	FERMT3	HIST1H2BM	ITGB2	NABP2	PTPN6	TCN1	
CASP1	CMAHP	FKBP11	HIST1H3J	KIF15	NBEAL2	RAB33A	TESC	
CCDC167	COMMD4	FLT3LG	HIST1H4B	KIF18B	NDC80	RAB37	TIMM10	
CCDC86	CORO1A	FOXM1	HIST1H4I	KIF20A	NEAT1	RACGAP1	TIMP1	
CCL3	COTL1	GABARAPI	HIST2H2AA3	KIF23	NKG7	RGS19	TMEM107	
CCL4	CREG1	GEMIN6	HIST2H2AA4	KIF4A	NME1	RNF167	TMPO	

B

Leukocyte Activation (27)

ARHGAP9
BATF
C3AR1
CCL3
CCL5
CEACAM1
CORO1A
COTL1
CSF1
CTSS
DPP4
FCER1G
FLT3LG
GLIPR1
GSTP1
IL4
IL13
ITGAX
ITGB2
LAIR1
LCK
MFNG
NBEAL2
PTPN6
RAB37
TCN1
TNFSF14

Leukocyte Migration (15)

C3AR1
CCL4
CCL5
CCR1
CCR5
CEACAM1
CORO1A
CXCR3
FCER1G
IL16
ITGAX
ITGB2
LCK
PTPN6
SLC7A5

Somatic Diversification of Immune Receptors (2)

BATF
HMGB2

Activation of Immune Response (9)

C3AR1
CTSS
FCER1G
HLA-DQA1
HLA-DRB5
IRAK1
ITGB2
LCK
PTPN6

Leukocyte Homeostasis (4)

CCNB2
CORO1A
MTHFD1
TNFSF14

Lymphocyte Costimulation (6)

DPP4
HLA-DQA1
HLA-DRB5
LCK
PTPN6
TNFSF14

Immune Effector Process (24)

APOBEC3B
ARHGAP9
BATF
C3AR1
CCL3
CD74
CEACAM1
CORO1A
COTL1
CTSS
FCER1G
GLIPR1
GSTP1
GZMB
IFITM1
IL4
ITGAX
ITGB2
LAIR1
MFNG
NBEAL2
PTPN6
RAB37
TCN1

Antigen Processing and Presentation (10)

CD74
CTSS
FCER1G
HLA-DQA1
HLA-DRB5
KIF4A
KIF15
KIF23
RAB33A
RACGAP1

Production of Molecular Mediator of Immune Response (1)

IL4

T cell Selection (1)

CD74

Myeloid cell Homeostasis (1)

MTHFD1

Immune Response (28)

APOBEC3B
CCL4
CCR1
CCR5
CD74
CORO1A
CSF1
CST7
CTSS
CTSW
FCER1G
GNLY
GZMA
GZMB
HLA-DQA1
HMGB2
IL4
IL13
IL16
LAIR1
LCK
LIF
NRROS
PPP1R14B
PTGDR2
PTPN6
TNFSF12
TNFSF14

C

Upregulated immune system genes

BIRC3
CREG1
EGR1
HEXIM1

Supplementary Figure S4

A Lymphoid cell gating strategy

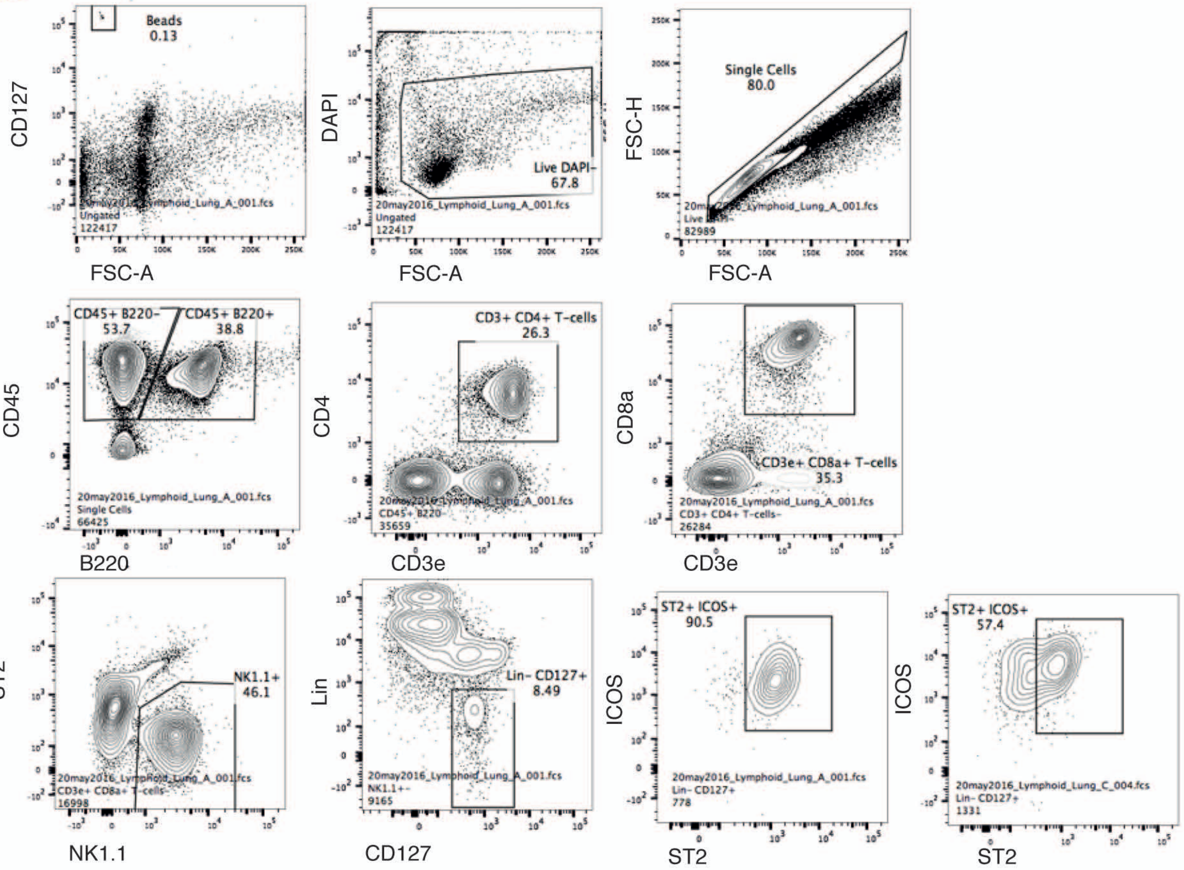

B Myeloid cell gating strategy

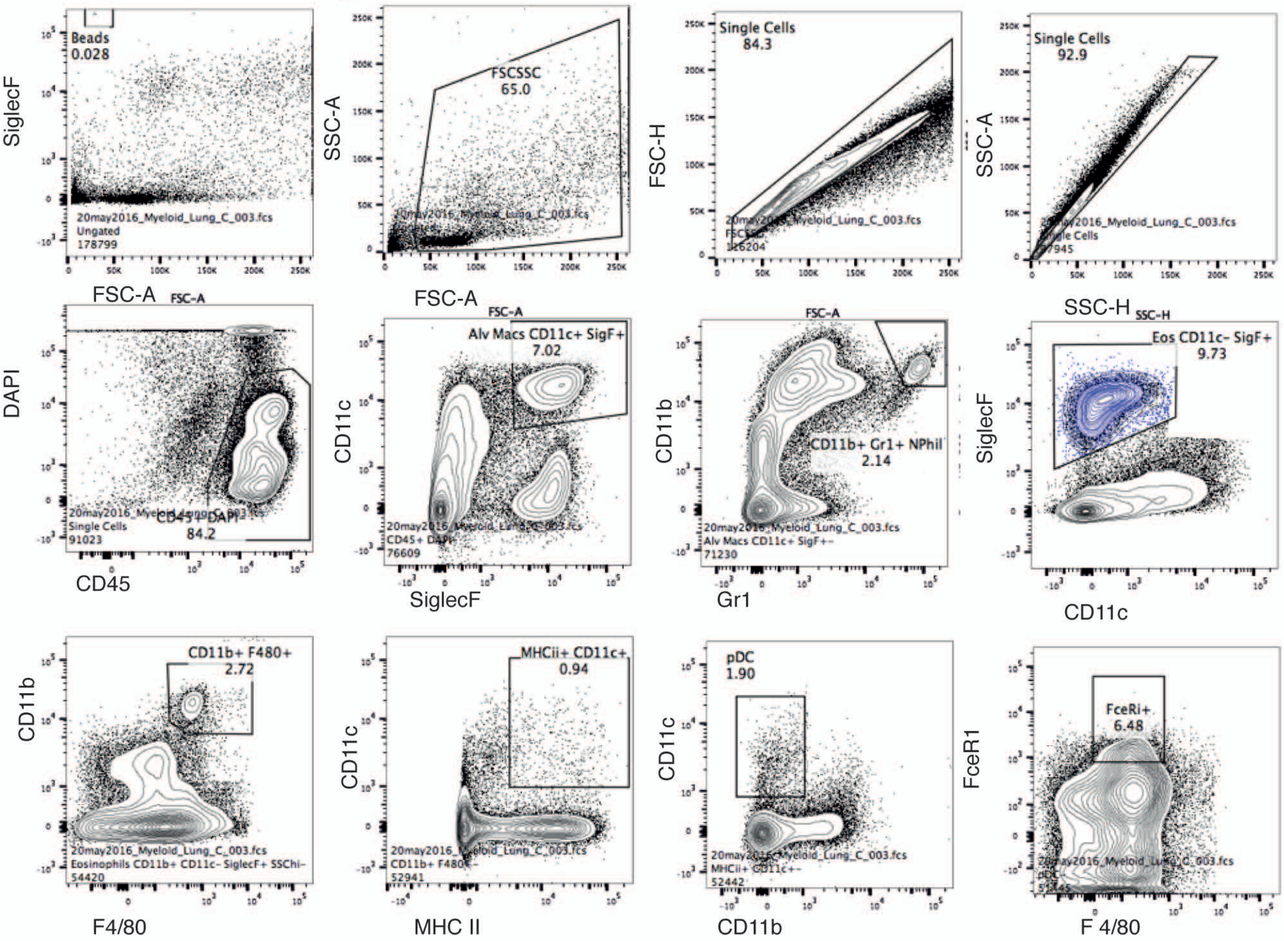

Supplementary Figure S5

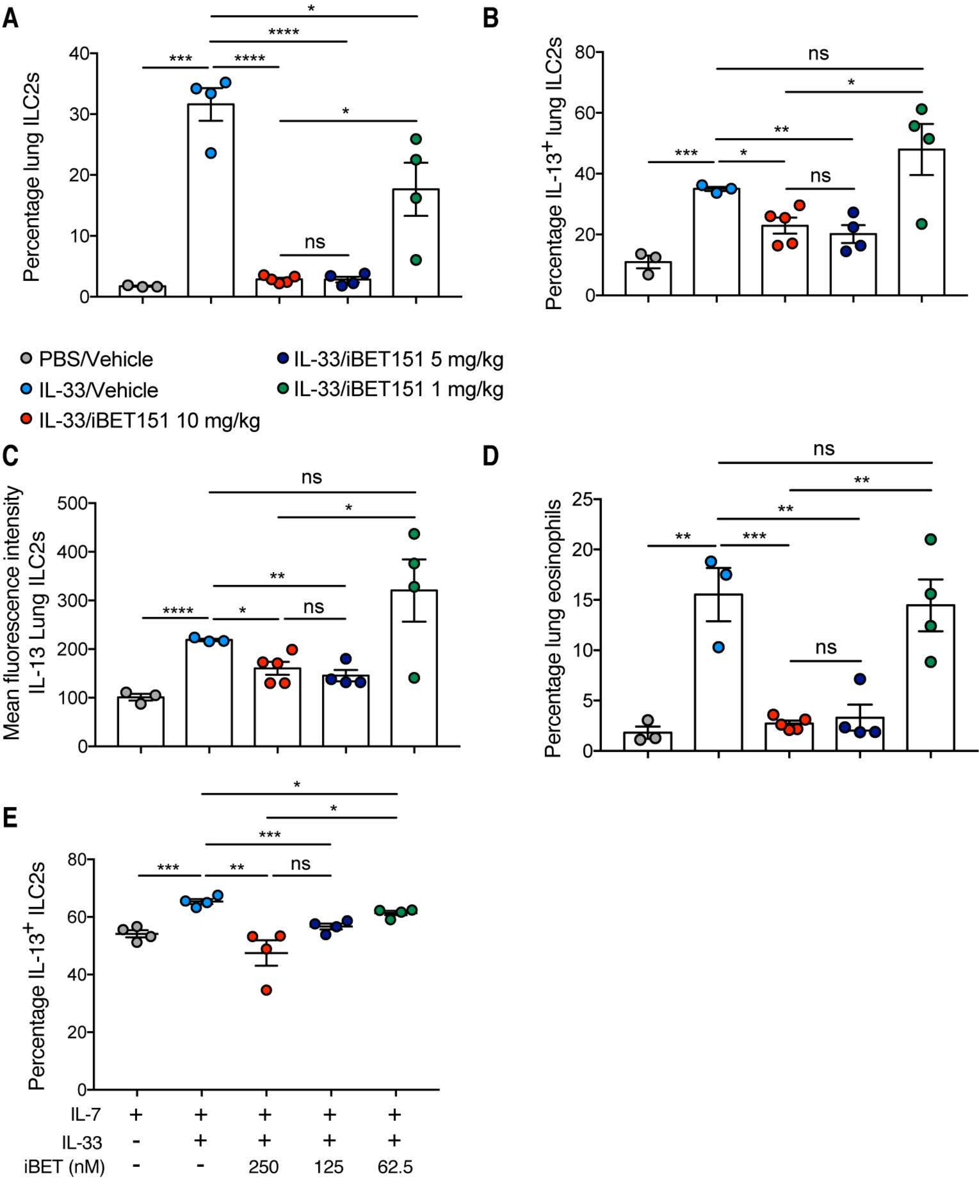

Supplement: Supplementary Figure S1 — Human blood ILC subsets analyzed by flow cytometry. [file Data_Sheet_1.PDF]
